# Development and validation of a preoperative radiomics-based nomogram to identify patients who can benefit from splenic hilar lymphadenectomy: a pooled analysis of three prospective trials

**DOI:** 10.1097/JS9.0000000000001337

**Published:** 2024-04-23

**Authors:** Bin-bin Xu, Hua-Long Zheng, Chun-sen Chen, Liang-liang Xu, Zhen Xue, Ling-hua Wei, Hong-hong Zheng, Li-li Shen, Chao-Hui Zheng, Ping Li, Jian-Wei Xie, Jian-xian Lin, Yu-hui Zheng, Chang-Ming Huang

**Affiliations:** a Department of Gastric Surgery, Fujian Medical University Union Hospital; b Department of Radiology, Fujian Medical University Union Hospital; c Department of Pathology, Fujian Medical University Union Hospital; d Key Laboratory of Ministry of Education of Gastrointestinal Cancer, Fujian Medical University; e Fujian Key Laboratory of Tumor Microbiology, Fujian Medical University; f Department of Radiology, Fuzhou Pulmonary Hospital of Fujian, Educational Hospital of Fujian Medical University; g Fujian Province Minimally Invasive Medical Center, Fuzhou, People’s Republic of China

**Keywords:** gastric cancer, pooled analysis, radiomic nomogram, recurrence, spleen-preserving splenic hilar lymphadenectomy

## Abstract

**Background:**

The authors aimed to use preoperative computed tomography images to develop a radiomic nomogram to select patients who would benefit from spleen-preserving splenic hilar (No.10) lymphadenectomy (SPSHL).

**Methods:**

A pooled analysis of three distinct prospective studies was performed. The splenic hilar lymph node (SHLN) ratio (sLNR) was established as the quotient of the number of metastatic SHLN to the total number of SHLN. Radiomic features reflecting the phenotypes of the primary tumor (RS1) and SHLN region (RS2) were extracted and used as predictive factors for sLNR.

**Results:**

This study included 733 patients: 301 in the D2 group and 432 in the D2+No.10 group. The optimal sLNR cutoff value was set at 0.4, and the D2+No.10 group was divided into three groups: sLNR=0, sLNR ≤0.4, and sLNR >0.4. Patients in the D2+No. 10 group were randomly divided into the training (*n*=302) and validation (*n*=130) cohorts. The AUCs value of the nomogram, including RS1 and RS2, were 0.952 in the training cohort and 0.888 in the validation cohort. The entire cohort was divided into three groups based on the nomogram scores: low, moderate, and high SHLN metastasis burden groups (LMB, MMB, and HMB, respectively). A similar 5-year OS rate was found between the D2 and D2+No. 10 groups in the LMB and HMB groups. In the MMB group, the 5-year OS of the D2+No. 10 group (73.4%) was significantly higher than that of the D2 group (37.6%) (*P*<0.001).

**Conclusions:**

The nomogram showed good predictive ability for distinguishing patients with various SHLN metastasis burdens. It can accurately identify patients who would benefit from SPSHL.

## Introduction

Highlights

- This study developed individualized radiomics-based nomogram for splenic hilar lymph node (SHLN) dissection.
- The nomogram showed good predictive ability for distinguishing patients with various SHLN metastasis burdens.
- It can accurately identify patients who would benefit from SHLN dissection.

Globally, gastric cancer (GC) ranks fifth in incidence and fourth in mortality among malignant tumors^1^. Gastrectomy with D2 lymph node (LN) dissection is the only curative approach for locally advanced GC. Consequently, safe and thorough clearance of LNs has become a prominent topic in the surgical management of GC. For patients with advanced proximal gastric cancer (APGC), LN in the splenic hilar lymph node (SHLN) are crucial components of the lymphatic drainage system.

Prior to the publication of the 5th edition of the ‘Japanese Gastric Cancer Treatment Guidelines (JGCT)’, it was stipulated that for patients with APGC who should undergo total gastrectomy, D2 lymphadenectomy should include clearance of the SHLN (No. 10 LN)^2^. However, based on the survival outcomes of the Japanese Clinical Oncology Group (JCOG) 0110 study^3^, the 5th edition of the guidelines removed the recommendation for routine SHLN dissection as part of D2 lymphadenectomy. Instead, it only recommends SHLN dissection (D2+No. 10) for patients with tumor involvement of the greater curvature^4^. Nevertheless, a single-center randomized controlled study has demonstrated that for patients with APGC located in the posterior wall of the stomach, spleen-preserving SHLN dissection (SPSHL) can provide survival benefits^5^. Furthermore, several studies have shown that apart from tumor involvement of the greater curvature, tumor size, and preoperative tumor staging are also associated with SHLN metastasis. Therefore, some researchers have suggested performing No. 10 lymphadenectomies in high-risk individuals^6,7^. However, some experts believe that when No. 10 LN metastasis is present, the prognosis is generally poor, considering No. 10 LN metastasis as an incurable factor and classifying it as distant metastasis^8,9^. Consequently, they advocated against routine No. 10 lymphadenectomy for patients at a high-risk of SHLN metastasis. To sum up, there is currently no consensus on which types of patients with GC should undergo No. 10 lymphadenectomy, and whether all high-risk patients with SHLN metastasis can benefit from SPSHL remains unclear.

Human cancers exhibit strong phenotypic differences that can be visualized noninvasively by medical imaging. The most widely used imaging modality in oncology is X-ray computed tomography (CT), which assesses tissue density. Although some investigations have characterized the appearance of a tumor on CT images, these characteristics are typically described subjectively and qualitatively (‘moderate heterogeneity’, ‘highly spiculated’, ‘large necrotic core’). Recent advances in medical imaging acquisition and analysis allow the high-throughput extraction of informative imaging features to quantify the differences that oncologic tissues exhibit in medical imaging^10^. Radiomics refers to the comprehensive quantification of tumor phenotypes by applying a large number of quantitative image features. Radiomics applies advanced computational methodologies to medical imaging data to convert medical images into quantitative descriptors of oncologic tissues. Radiomic features are first-order or higher-order metrics that capture quantitative information such as tumor image intensity, shape, texture, and multiscale wavelet within the imaging data^11,12^.

Previous data showed that radiomics, which extracted multidimensional quantitative data from standard medical imaging, could accurately predict cancer dissemination and prognosis^13,14^. In the study of Dong *et al*.^15^, the authors delineated both the tumor and peritoneal regions, and studied the characteristics on CT images to reflect the peritoneal microenvironment, ultimately achieving the purpose of detecting peritoneal metastasis. Li *et al*.^16^ delineated the complete lesions of intestinal Crohn’s disease, and extracted the features on the CT images to achieve the purpose of determining the degree of local fibrosis of the lesions. Mukherjee *et al*.^17^ also detected and quantified the imaging signature of early pancreatic carcinogenesis from whole volumetrically segmented normal pancreas on CTs to constructed a predictive model that can identify early pancreatic cancer.

To date, no studies have applied radiomics techniques to distinguish patients who would benefit from spleen-preserving SHLN dissection. Therefore, we hypothesized that the radiomics features from the center and contour of the tumor and splenic hilar regions of interest are excellent noninvasive biomarkers to predict the metastatic burden of SHLN.

Therefore, we included data from three prospective clinical trials for a pooled analysis and constructed the ‘splenic hilar lymph node ratio’ (sLNR) as an indicator to reflect the extent of No. 10 LN metastasis. We aimed to identify the true beneficiaries of SPSHL and utilize radiomics to develop a nomogram that can be applied preoperatively to differentiate patients with different levels of SHLN metastatic burden and provide surgeons with evidence-based guidance for individualized surgical interventions.

## Materials and methods

### Patients

From January 2015 to July 2019, 1225 patients were enrolled in three independent prospective trials, including the CLASS-04 trial^18^, FUGES-001 trial^19^, and FUGES-002 trial^5^. The detailed information about the three trials were previously reported and provided in Supplementary Materials (Supplemental Digital Content 1, http://links.lww.com/JS9/C427). Patients who underwent curative total gastrectomy were eligible for the pooled analysis. The exclusion criteria consisted of the following: patients who withdrew their consent, intraoperatively confirmed inability to achieve R0 resection owing to tumor factors, those who underwent partial gastrectomy, individuals with tumors in the lower third of the stomach, and cases involving tumors invading the esophagus (as demarcated by the dentate line connecting the esophagus and stomach). Herein, the patients were divided into two groups: D2 and D2+No. 10 group, as previously described^6,7^. The detailed surgical procedures were described in Supplementary Materials. Fig. S1 (Supplemental Digital Content 3, http://links.lww.com/JS9/C429) shows the intraoperative view of the splenic hilum after D2+No. 10 lymph node dissection. This study was approved by the Institutional Review Board. This study has been reported in line with the STROCSS criteria^20^. The video demonstrates the procedure of LSPSHL (Supplement video Supplemental Digital Content 12, http://links.lww.com/JS9/C467).

### Definitions

The clinical T (cT) and N (cN) disease categories were classified according to the 7th edition of the American Joint Committee on Cancer (AJCC) staging manual^21^.

The SHLN ratio was used to reflect the burden of No. 10 LNs metastasis and was defined as the ratio of positive No. 10 LNs to the total No. 10 LN harvest.

We calculated the therapeutic value index (TVI) reflect the value of LN dissection^22^. The index was determined by multiplying the frequency of metastatic nodal stations with the 5-year overall survival (OS) rate observed in patients with such metastatic nodal stations.

OS was defined as the time interval from the date of surgery to the date of death or last follow-up, while disease-free survival (DFS) was defined as the time interval from the date of surgery to either the date of recurrence or death from any cause.

Recurrence was categorized by site as follows: locoregional, peritoneal, distant, or multiple^23–25^. Multiple recurrences were defined as the occurrence of disease recurrence at two or more distinct sites.

### Image acquisition, processing, and feature extraction

In the present study, patients underwent contrast-enhanced abdominal CT scans before therapy. Portal venous-phase CT images were obtained using a picture archiving and communication system. The median (range) CT slice thickness was 1.25 (0.625–5) mm. The three-dimensional volumes of interest (VOI) of the tumor (VOI-1) and splenic hilar (VOI-2) areas were manually segmented independently on the venous-phase CT images by two radiologists (CS.C. and LL.X.) who are experienced with the field. The VOIs were drawn along the contours of the target regions on each transverse section until the full lesion was captured using 3D Slicer version 5.0.3. Radiologists were blinded to the pathological results and patient outcomes. Details regarding the image feature extraction were described in the Supplementary Materials (Supplemental Digital Content 1, http://links.lww.com/JS9/C427).

### Radiomic feature selection and signature building

The radiomics features of all patients were standardized using the z-score method based on the parameters calculated from the training cohort. Feature selection and signature building processes were carried out on both the primary tumor and splenic hilar areas, including three steps: (i) features with high stability (intraclass correlation coefficient >0.80) between two readers were retained for further analysis; (ii) features that were potentially biased by CT slice thickness were excluded; detailed descriptions are presented in the Supplementary Materials (Supplemental Digital Content 1, http://links.lww.com/JS9/C427); (iii) The least absolute shrinkage and selection operator Method (LASSO) logistic regression method was used to identify the most predictive features of No. 10 LN metastasis. Subsequently, radiomic signatures reflecting the primary tumor (RS1) and splenic hilar area (RS2) were built as predictors of sLNR. The detailed formulas for the calculation of RS1 and RS2 are presented in the Supplementary Materials (Supplemental Digital Content 1, http://links.lww.com/JS9/C427).

### Statistical analysis

Multivariable logistic regression analysis was performed to select the independent predictors of No. 10 LN metastasis. We developed a radiomic nomogram with radiomic features and a clinical model only including clinical characteristics for comparison. The accuracy of the two models was evaluated by receiver operating characteristic (ROC) curves. The radiomics nomogram was then calibrated using a calibration curve. Decision curve analysis was conducted to assess the clinical usefulness of the two models. The optimal cutoff values of the nomogram score for distinguishing patients into different risk groups were obtained using the ROC curve. Statistical analyses were performed using SPSS v.18.0 for Windows (SPSS Inc.) and R (http://www.r-project.org). Statistical significance was set at *P*<0.05.

## Result

### Characteristics of the included cohorts

**Figure 1:**
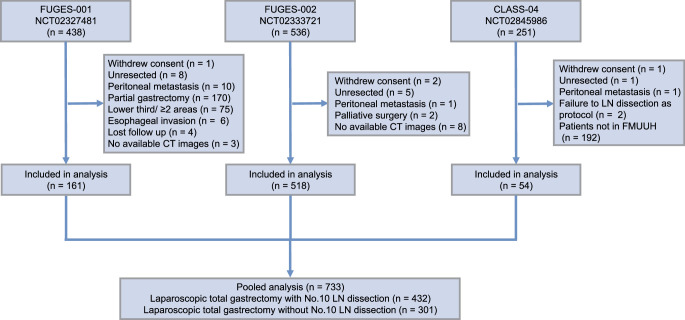
Flow chart of the trial.

The initial cohort consisted of 1225 patients, out of which 733 patients were finally included (Fig. 1). Among them, 301 patients underwent D2 lymphadenectomy, and 432 patients underwent D2+No. 10 lymphadenectomy. Median follow-up duration for the cohort was 67 months (95% CI: 64–70 months). Among the included patients, there were 541 men (73.8%) and 192 women (26.2%), with a mean age of 60.3 years (49.0–64.0). Most patients (*n*=620, 84.6%) had pathological stage II or III disease. The other baseline characteristics were summarized in Table 1.

**Table 1:** Characteristics of included patients.

	D2 Group, No. (%)	D2 + No.10 Group, No. (%)	
	*n*=301	*n*=432	*P*
Age, years			0.545
≤60	118 (39.2%)	179 (41.4%)	
>60	183 (60.8%)	253 (58.6%)	
Sex			0.093
Male	232 (77.1%)	309 (71.5%)	
Female	69 (22.9%)	123 (28.5%)	
cT stage			0.494
≤cT2	58 (19.3%)	98 (22.7%)	
cT3	154 (51.2%)	206 (47.7%)	
cT4	89 (29.6%)	128 (29.6%)	
cN stage			0.182
cN0	81 (26.9%)	136 (31.5%)	
cN+	220 (73.1%)	296 (68.5%)	
Tumor size, cm			<0.001
≤5	163 (54.2%)	301 (69.7%)	
>5	138 (45.8%)	131 (30.3%)	
Histology			0.207
Differentiated	106 (35.2%)	172 (39.8%)	
Undifferentiated	195 (64.8%)	260 (60.2%)	
Cross-sectional part			0.053
Nongreater curvature	282 (93.7%)	387 (89.6%)	
Greater curvature	19 (6.3%)	45 (10.4%)	
Lymphovascular invasion			0.096
Absent	169 (56.1%)	269 (62.3%)	
Present	132 (43.9%)	163 (37.7%)	
Perineural invasion			0.486
Absent	184 (61.1%)	253 (58.6%)	
Present	117 (38.9%)	179 (41.4%)	
pT stage			0.126
T1	29 (9.6%)	58 (13.4%)	
T2	25 (8.3%)	37 (8.6%)	
T3	148 (49.2%)	226 (52.3%)	
T4	99 (32.9%)	111 (25.7%)	
pN stage			0.001
N0	65 (21.6%)	134 (31.0%)	
N1	34 (11.3%)	77 (17.8%)	
N2	82 (27.2%)	82 (19.0%)	
N3a	81 (26.9%)	91 (21.1%)	
N3b	39 (13.0%)	48 (11.1%)	
pTNM stage			<0.001
I	41 (13.6%)	72 (18.7%)	
II	57 (18.9%)	133 (30.8%)	
III	203 (67.4%)	227 (52.5%)	
Adjuvant chemotherapy			0.589
Absent	104 (34.6%)	141 (32.6%)	
Present	197 (65.4%)	291 (67.4%)	

LNs, lymph nodes.

### Survival outcomes of patients with D2+No. 10

In the D2+No. 10 group, patients with SHLN metastasis had a significantly worse 5-year OS compared to those without No. 10 LN metastasis (5-year OS: 72.5 vs. 44.7%, *P*<0.001). Consistent results were observed for 5-year DFS (eFigure2A-B, Supplemental Digital Content 4, http://links.lww.com/JS9/C430 ). Subsequently, the sLNR was calculated based on the definition described in the Methods section. To identify the optimal cutoff value of sLNR for prognosis, we performed a stepwise analysis of sLNR cutoff values from 0.1 to 0.9 with an increment of 0.1 (eFigure3, Supplemental Digital Content 5, http://links.lww.com/JS9/C431). We found that when the sLNR cutoff value was set to 0.4, as shown in eFigure3 (Supplemental Digital Content 5, http://links.lww.com/JS9/C431) the OS of the sLNR=0 group was comparable to that of the sLNR ≤0.4 group (*P*=0.698), suggesting that patients in these groups achieved similar long-term outcomes after undergoing SPSHL. However, the sLNR >0.4 group had significantly worse long-term prognosis than the sLNR ≤0.4 group (*P*<0.001), indicating that patients with a high sLNR may not benefit from SPSHL. Furthermore, the ‘Maxstat’ function in R also confirmed that the greatest OS difference between the two sLNR groups was observed when the cutoff value was set at 0.4 (eFigure4, Supplemental Digital Content 6, http://links.lww.com/JS9/C432). Therefore, we divided the patients into three groups: sLNR=0, sLNR ≤0.4, and sLNR >0.4. Multivariable Cox regression analysis, adjusting for confounding factors such as age, tumor size, and tumor stage showed that sLNR >0.4 was associated with OS (HR: 1.808, 95% CI: 1.052–3.105), while sLNR ≤0.4 was comparable to sLNR=0 (HR: 0.826, 95% CI: 0.396–1.721) (eTable1, Supplemental Digital Content 2, http://links.lww.com/JS9/C428 ). Patients with sLNR >0.4 were older and exhibited more advanced clinical T stage compared to patients with sLNR ≤0.4 (eTable2, Supplemental Digital Content 2, http://links.lww.com/JS9/C428 ).

### Feature selection and radiomic signature building

Patients in the D2+No. 10 group were randomly divided into the training (*n*=302) and validation (*n*=130) cohorts in a 7:3 ratio. No significant differences in characteristics was observed between the two groups (eTable3, Supplemental Digital Content 2, http://links.lww.com/JS9/C428). In the training cohort, VOI-1 and VOI-2 were delineated using the 3Dslicer software (eFigure5, Supplemental Digital Content 7, http://links.lww.com/JS9/C433 ), and 1130 features were extracted from each region. After evaluating the repeatability and eliminating variables related to slice thickness, 721 features from VOI-1 and VOI-2 were included for further analysis. Subsequently, LASSO regression analysis (eFigureS6, Supplemental Digital Content 8, http://links.lww.com/JS9/C434 ) identified four features from VOI-1 and five features from VOI-2 as two radiomic signatures: RS1 and RS2 (eTable4, Supplemental Digital Content 2, http://links.lww.com/JS9/C428). The radiomics scores (RS1 and RS2) showed significant differences among the different sLNR groups (eFigure7A-D, Supplemental Digital Content 9, http://links.lww.com/JS9/C435).

### Development and validation of a radiomic nomogram

**Figure 2:**
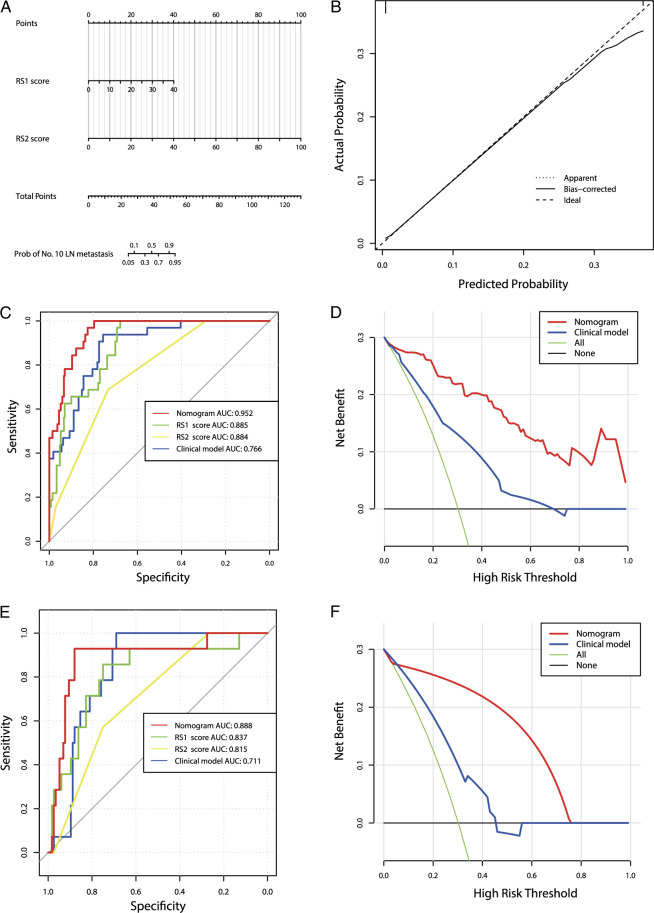
Development and performance of different models. (A) Nomogram based on radiomic signatures. (B) Calibration curves of the radiomic nomogram in the training cohort. ROC curves and decision curve analysis in the training cohort (C,D) and validation cohorts (E,F).

Multivariate analysis of clinical characteristics and radiomics scores showed that only RS1 and RS2 were associated with No. 10 LN metastasis. When excluding radiomics features, tumor size >5 cm, tumor located in the greater curvature of the stomach, and cN stage were independent predictors of No. 10 LN metastasis (Table 2). Thus, a nomogram that included only radiomics scores and another clinical model that included only clinical features were developed. Calibration curves of the nomogram showed good consistency between the predicted and observed values across all cohorts. In addition, the nomogram had higher area under the curve (AUC) values in the training (0.952) and validation cohorts (0.888) than the clinical model and RS1 and RS2 alone (*P*<0.05). Decision curves were used to compare the net benefits of the two models. At any given threshold probability, the nomogram had higher net benefits for intervention decision-making than the clinical model (Fig. 2A-F, eFigure8A-B, Supplemental Digital Content 10, http://links.lww.com/JS9/C436 ).

**Table 2:** Risk clinical factor analysis of the no. 10 lymph node metastasis for patients with no. 10 LN dissection.

	Univariate analysis		Multivariate analysis with RS scores		Multivariate analysis without RS scores	
	OR (95% CI)	*P*	OR (95% CI)	*P*	OR (95% CI)	*P*
Age, years						
≤60	1.000					
>60	1.374 (0.646–2.922)	0.409				
Sex						
Male	1.000					
Female	0.913 (0.405–2.059)	0.826				
Histologic type						
Differentiated	1.000					
Undifferentiated	0.756 (0.361–1.586)	0.460				
RS1 score	1.249 (1.169–1.334)	<0.001	1.245 (1.132–1.370)	<0.001	—	—
RS2 score	1.238 (1.158–1.323)	<0.001	1.224 (1.128–1.327)	<0.001	—	—
Tumor size, cm						
≤5	1.000		1.000		1.000	
>5	3.731 (1.756–7.927)	0.001	0.548 (0.161–1.862)	0.335	2.429 (1.070–5.512)	0.034
Cross-sectional part						
Nongreater curvature	1.000		1		1	
Greater curvature	4.011 (1.668–9.643)	0.002	2.044 (0.581–7.194)	0.265	2.840 (1.084–7.436)	0.034
cT stage						
≤cT2	1.000		1.000		1.000	
cT3	1.326 (0.347–5.069)	0.680	0.164 (0.022–1.202)	0.075	0.309 (0.064–1.492)	0.144
cT4	5.728 (1.623–20.209)	0.007	0.267 (0.036–1.981)	0.196	0.905 (0.194–4.211)	0.899
cN stage						
cN0	1.000		1.000		1.000	
cN+	17.663 (2.374–131.391)	0.005	6.944 (0.761–63.384)	0.086	16.936 (1.890–151.798)	0.011

RS, radiomics signatures.

### Survival benefit analysis of SPSHL

**Figure 3:**
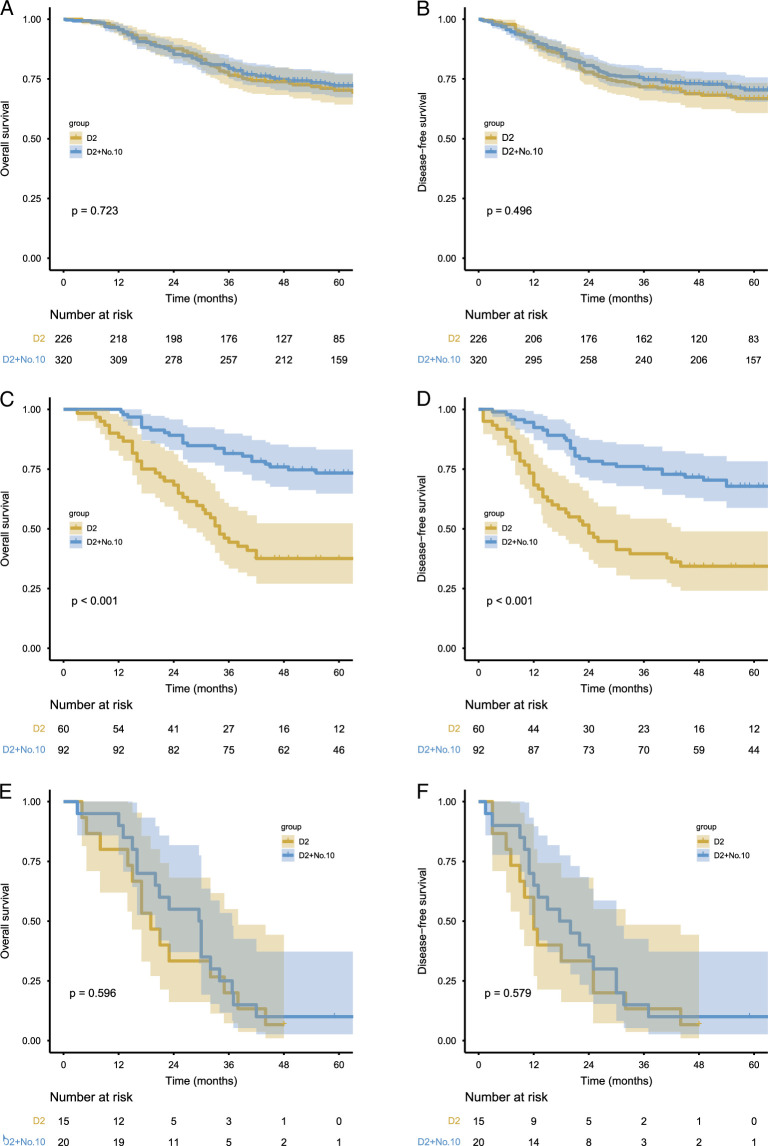
Comparing the effects of splenic hilar lymphadenectomy on OS and DFS under different splenic hilar lymph node (SHLN) metastasis burden. (A–B) low SHLN metastasis burden; (C–D) moderate SHLN metastasis burden; (E–F) high SHLN metastasis burden.

Risk scores for SHLN metastasis were calculated for all patients using a nomogram formula. Subsequently, the optimal cutoff point for distinguishing the sLNR was obtained from the ROC curve. The patients were then divided into three groups: low No. 10 LN metastasis burden (LMB) (score<25), moderate No. 10 LN metastasis burden (MMB) (score 25-43), and high No. 10 LN metastasis burden (HMB) (score>43). The No. 10 LN metastasis rate and No. 10 LN TVI for each group are shown in eTable5 (Supplemental Digital Content 2, http://links.lww.com/JS9/C428). Survival curves showed that for patients in the LMB group, the 5-year OS of the D2 group was similar to that of the D2+No. 10 group (70.3 vs. 72.1%, *P*=0.723), with a TVI of 0.6. In the MMB group, the 5-year OS of the D2+No. 10 group was significantly higher than the 5-year OS of the D2 group (73.4 vs. 37.6%, *P*<0.001), with a TVI of 18.0. In the HMB group, both the two groups had poor prognosis with similar 5-year OS (6.7 vs. 10.0%, *P*=0.723), with a TVI of 5.0. Similar results were observed in the DFS (Fig. 3A-F). Multivariate analysis revealed that SPSHL was associated with better OS only in the MMB group. However, in the LMB and HMB groups, SPSHL was not associated with prolonged prognosis. When comparing the postoperative recurrence patterns among the different groups, it was observed that the performance of SPSHL did not affect the postoperative recurrence patterns in the LMB or HMB groups. Contrastingly, in the MMB group, regardless of overall recurrence, local recurrence, distant metastasis, or multiple-site metastasis, the rate of disease recurrence in the D2+No. 10 group was found to be significantly lower compared to the D2 group (*P*<0.05) (eFigure9A-C, Supplemental Digital Content 11, http://links.lww.com/JS9/C437).

## Discussion

We collected data from three prospective trials and established a radiomics nomogram that serves as an easily accessible and personalized tool for distinguishing patients with different No. 10 LN metastasis burdens. This tool utilizes only preoperative CT image data and aids surgeons in determining optimal candidates for SPSHL prior to surgery.

In recent years, there has been a growing body of research dedicated to investigating No. 10 LN metastases. Shin *et al*.^8^ performed analyses involving 319 patients with APGC and found that although the No. 10 LN metastasis rate was 12.9%, no patients with No. 10 LN metastasis were identified among patients with early GC. The 4th edition of the JGCT explicitly stated that for patients with APGC who require total gastrectomy, standard D2 lymphadenectomy should include No. 10 LN. However, based on the survival results of the JCOG 0110 study, the 5th edition of the JGCT removed the SHLN dissection from the D2 lymphadenectomy and only recommended performing SHLN dissection for patients with tumors invading the greater curvature. Several studies have indicated that the presence of SHLN metastasis is associated with factors such as tumor location, size, infiltration depth, presence of greater curvature, and Borrmann classification^6,7,26^. However, in this study, it was found that when radiomics features were incorporated into multivariate analysis, they were closely correlated with No. 10 LN metastases. Additionally, the radiomics nomogram demonstrated significantly better predictive performance than the clinical models. This may be attributed to the 3D volume delineation of the primary tumor and splenic hilar regions in the original CT images, which allowed the selected radiomics features to better reflect tumor characteristics than clinical variables, such as tumor size and cN staging.

Since the first report by Hyung in 2008 on laparoscopic SPSHL (LSPSHL), more and more studies have demonstrated the safety and feasibility of LSPSHL as well as its advantages in preserving organ function and promoting rapid recovery^18,27,28^. Consequently, LSPSHL is now widely employed. However, owing to the deep and narrow location of the splenic hilum, and the complex and variable course of the splenic vessels, implementing LSPSHL remains a challenge for surgeons. Moreover, several studies have shown that patients with SHLN metastasis have a significantly worse prognosis than those without metastasis and that performing D2+No. 10 lymphadenectomy in high-risk metastasis subgroups can effectively improve their survival^6,7,29^. Maezawa *et al*. conducted a retrospective analysis of 82 patients with APGC invading the greater curvature. The results showed a high metastasis rate of SHLN, reaching 13.4%, with a TVI similar to that of LNs in the suprapancreatic areas. Therefore, the authors suggested that D2 lymphadenectomy for APGC invading the greater curvature should include SHLN dissection^29^. In contrast, Yoon *et al*.^30^ indicated that patients with APGC, even those with tumor involvement in the greater curvature, did not require SHLN dissection. Some scholars also believe that when SHLN metastasis is positive, patients tend to have a poor prognosis. Therefore, SHLN metastasis should be classified as distant metastasis, and routine SHLN dissection should not be performed in high-risk patients with No. 10 LN metastasis^8,9,31^. A single-center randomized controlled study demonstrated that No. 10 LN dissection only improved the prognosis of patients with tumors located on the posterior wall^5^. Therefore, not all patients with No. 10 LN metastases benefit from splenic hilar lymphadenectomy. Surgeons must carefully select patients who will benefit from LSPSHL and minimize any potential harm to those who will not benefit from the procedure.

Based on the decision tree classification, Zhong *et al*.^6^ developed a classification tool to differentiate the risk of No. 10 LN metastasis. However, the survival analysis included only 164 patients, which is a relatively small sample size. In this study, we constructed an innovative index, the sLNR, to reflect the burden of SHLN metastasis. Survival analysis revealed that patients with different sLNRs had varying survival benefits from SPSHL. Subsequently, we developed an imaging-based nomogram to preoperatively assess the burden of No. 10 LN metastasis, which performed well in both the training and validation cohorts. Using the formula calculated from this nomogram, we divided the entire patient cohort into different groups to reflect the burden of No. 10 LN metastases. We found that for patients with moderate No. 10 LN metastasis burden, a SHLN metastasis rate approaching 30% undergoing SPSHL resulted in significant survival benefits and effectively reduced the risk of postoperative recurrence. However, for individuals with a high burden of No. 10 LN metastasis, SPSHL did not provide any benefit owing to poor prognosis. Future research is warranted to further explore optimal treatment methods, such as neoadjuvant chemotherapy, for this population.

The current study had several limitations. First, although this pooled study provided a large amount of reliable real-world data, the three included clinical trials had their distinct research focuses, which may have influenced the results. Second, this study was conducted at a single-center, and the predictive performance of the nomogram requires further validation by external centers. Finally, in our study, delineation of the primary tumor and splenic hilar regions was performed by radiologists, which is a time-consuming and labor-intensive process. Considering the emergence of artificial intelligence technology, similar delineation work may be automated in the future and incorporated into the radiomics model used in this study. Nevertheless, to our knowledge, this study is the first to use radiomic techniques to accurately select appropriate candidates for splenic hilar lymphadenectomy, providing a basis for preoperative decision-making by surgeons. Regarding the challenging technique of SPSHL, patients who require SPSHL should not be deprived of the opportunity for radical treatment owing to technical difficulties, while avoiding the additional risk of harm to patients who do not benefit from the procedure.

## Conclusion

We established an indicator that can effectively distinguish the metastatic burden of SHLNs and developed a radiomics nomogram to accurately predict it using preoperative CT images. This tool can help guide surgeons in the preoperative screening of populations that may benefit from SPSHL and avoid additional damage to patients. Moreover, direct radical surgery may not be optimal for patients with a high SHLN metastasis burden.

## Ethical approval

This study was approved by the Institutional Review Board of Fujian Medical University Union Hospital (IRB number: 2024KY030) and, where applicable, followed the STROCSS criteria and guidelines.

## Consent

The need for informed consent was waived due to the retrospective nature of the study.

## Source of funding

This study was supported by the Fujian Province Medical ‘Dual High’ Construction Funding (Min Wei Yi Zheng No. [2021] 76).

## Author contribution

X.B.B., Z.H.L., H.C.M., and L.J.X.: concept and design and drafting of the manuscript; X.B.B. and Z.H.L.: statistical analysis; H.C.M.: obtained funding; C.C.S., X.L.L., and Z.Y.H.: administrative, technical, or material support; X.B.B., Z.H.L., H.C.M., and L.J.X.: supervision. All authors read and approved the final manuscript and contributed in acquisition, analysis, or interpretation of data, critical revision of the manuscript for important intellectual content.

## Conflicts of interest disclosure

There are no conflicts of interest or financial ties to disclose from any authors.

## Research registration unique identifying number (UIN)

1. Name of the registry: ClinicalTrials.gov.
2. Unique identifying number or registration ID: CLASS-04 study, ClinicalTrials.gov Identifier NCT02845986 FUGES-001 study, ClinicalTrials.gov Identifier NCT02327481 FUGES-00w study, ClinicalTrials.gov Identifier NCT02333721
3. Hyperlink to your specific registration (must be publicly accessible and will be checked): https://www.clinicaltrials.gov/study/NCT02845986. https://www.clinicaltrials.gov/study/NCT02327481 https://www.clinicaltrials.gov/study/NCT02333721

## Guarantor

Huang CM had full access to all the data in the study and takes responsibility for the integrity of the data and the accuracy of the data analysis.

## Data availability statement

The datasets used and/or analyzed during the current study are available from the corresponding author on reasonable request.

Huang CM had full access to all the data in the study and takes responsibility for the integrity of the data and the accuracy of the data analysis. De-identified data about the individual participants will be shared with researchers of further studies on reasonable request. Request for data sharing will be handled in line with the data access and sharing policy of Fujian Medical University Union Hospital.

## Provenance and peer review

Not commissioned, externally peer-reviewed.

## Supplementary Material

SUPPLEMENTARY MATERIAL
