# Supplementary material for: Development and validation of a preoperative radiomics-based nomogram to identify patients who can benefit from splenic hilar lymphadenectomy: a pooled analysis of three prospective trials
Source: Int J Surg. 2024 Apr 23;110(7):4053–61. doi: 10.1097/JS9.0000000000001337 (PMC11254245; doi:10.1097/JS9.0000000000001337)
Supplement: SUPPLEMENTARY MATERIAL [file js9-110-4053-s001.docx]

**Supplementary Materials**

**Patients from three prospective trials**

From January 2015 to July 2019, 1240 patients were enrolled in three independent prospective trials. The CLASS-04 trial (ClinicalTrials.gov, NCT02845986) enrolled 251 patients between September 14, 2016, and October 12, 2017, to evaluate the safety and feasibility of laparoscopic spleen-preserving splenic hilar lymphadenectomy (LSPHSL) for advanced proximal gastric cancer (APGC), conducted at specialized institutions of the Chinese Laparoscopic Gastrointestinal Surgery Study group^[1](#_ENREF_1)^. The FUGES-001 trial (ClinicalTrials.gov, NCT02327481) enrolled 438 patients between January 1, 2015, and April 1, 2016, to determine the relative safety and eﬃcacy of 3D and 2D laparoscopic gastrectomies in GC, conducted at Fujian Medical University Union Hospital (FMUUH)^[2](#_ENREF_2)^. The FUGES-002 trial (ClinicalTrials.gov, NCT02333721), conducted at FMUUH, enrolled 536 patients between January 5, 2015, and December 10, 2018, to evaluate the surgical outcomes of LSPHSL for APGC without invading the greater curvature^[3](#_ENREF_3)^. All three studies were approved by the local ethics committee. Operative techniques, perioperative management, definitions of study endpoints, and results of these studies have been previously reported. All three trials had similar inclusion and exclusion criteria, except for definite tumor location and clinical T stage in each protocol. All the patients underwent the same perioperative management and follow-up protocol.

**Surgical procedures**

**D2 group:** laparoscopic total gastrectomy (LTG) with D2 lymphadenectomy. The extent of lymphadenectomy in the D2 group included the Nos. 1, 2, 3, 4, 5, 6, 7, 8a, 9, 11p, 11d, and 12a LNs.

**D2+No. 10 group:** LTG with D2 plus SPSHL. The extent of lymphadenectomy in the D2+No. 10 group included the Nos. 1, 2, 3, 4, 5, 6, 7, 8a, 9, 10, 11p, 11d, and 12a LNs. No. 10 LN included those adjacent to the splenic artery distal to the pancreatic tail, those on the roots of the short gastric arteries, and those along the left gastroepiploic artery proximal to the first gastric branch. During lymphadenectomy, the pancreas and spleen were not mobilized. The left gastroepiploic artery was ligated and cut at its origin, and the LNs along the splenic artery and hilum of the spleen were dissected without sacrificing the spleen and splenic vessels.^[4](#_ENREF_4)^

**Image feature extraction**

Image features at different spatial scales were derived using a Laplacian of Gaussian spatial band-pass filter (∇2G), with the filter parameter ranging from 1.5 to 2.5 (1.5, 2.0, 2.5).^[5](#_ENREF_5)^ Finally, two groups of features (1130 each) were extracted from VOI-1 and VOI-2. These features included four groups [18]: (i) first-order statistics: n = 18; (ii) shape and size features: n = 14; (iii) textural features derived from texture matrices including grey-level co-occurrence matrix (glcm), grey-level run length matrix (glrlm), grey-level size zone matrix (glszm), grey-level dependence matrix (gldm), neighbourhood gray-tone difference matrix (ngtdm): n = 75; and (iv) filter-derived features: filter “wavelet”: n = 744; filter ‘log’: n = 279.

**Radiomic feature selection and signature building**

To reduce the potentially confounding effect of slice thickness on the predictive models, we used a recently described process to identify and then account for potential dependence of the extracted features on slice thickness^[6](#_ENREF_6), [7](#_ENREF_7)^: The identification of radiomics features dependent on slice thickness involved three steps. First, all CTs were divided into 2 groups: slice thickness ≥3 mm and slice thickness <3mm. The selection of 3 mm as a cutoff was based on the current clinical practice. Second, AUC was calculated based on the slice thickness group labels for each feature with high stability (intraclass correlation coefficient >0.80). In other words, we attempted to measure how much of the radiomics feature was explained by the CT slice thickness. The AUC provided a goodness-of-fit measure of a given feature with the binary outcome (groups of slice thickness≥3 mm and <3 mm). Third, features with a high AUC (> 0.8) were deemed to be heavily influenced/biased by CT slice thickness and were, therefore, removed.

**The calculation formula of the RS1 and RS2**

Radiomic signature reflecting the features of the primary tumor area (RS1) and another radiomic signature reflecting that of splenic hilar area (RS2) were built as predictors of splenic hilar lymph node ratio (sLNR).

The calculation formula of the RS1：0.105960275 + 0.019888981* log-sigma-2-0-mm-3D_gldm_LargeDependenceHighGrayLevelEmphasis + 0.016135357* wavelet-LHH_glszm_LargeAreaHighGrayLevelEmphasis -0.008789638*wavelet-HLL_glcm_Imc2 + 0.041629276* wavelet-HHH_firstorder_Kurtosis

The calculation formula of the RS2：0.10596029 + 0.0260577* log-sigma-1-5-mm-3D_glcm_InverseVariance + 0.0248952* log-sigma-2-5-mm-3D_firstorder_Kurtosis + 0.03872172*wavelet-LHH_firstorder_Skewness + 0.06053678* wavelet-HHL_glcm_ClusterProminence + 0.03336477* wavelet-HHH_firstorder_Kurtosis

**Reference**

1. Zheng C, Xu Y, Zhao G, et al. Outcomes of Laparoscopic Total Gastrectomy Combined With Spleen-Preserving Hilar Lymphadenectomy for Locally Advanced Proximal Gastric Cancer: A Nonrandomized Clinical Trial. *JAMA Netw Open* 2021; 4(12):e2139992.

2. Zheng CH, Lu J, Zheng HL, et al. Comparison of 3D laparoscopic gastrectomy with a 2D procedure for gastric cancer: A phase 3 randomized controlled trial. *Surgery* 2018; 163(2):300-304.

3. Lin JX, Lin JP, Wang ZK, et al. Assessment of Laparoscopic Spleen-Preserving Hilar Lymphadenectomy for Advanced Proximal Gastric Cancer Without Invasion Into the Greater Curvature: A Randomized Clinical Trial. *JAMA Surg* 2023; 158(1):10-18.

4. Huang CM, Chen QY, Lin JX, et al. Huang's three-step maneuver for laparoscopic spleen-preserving No. 10 lymph node dissection for advanced proximal gastric cancer. *Chin J Cancer Res* 2014; 26(2):208-10.

5. Huang W, Jiang Y, Xiong W, et al. Noninvasive imaging of the tumor immune microenvironment correlates with response to immunotherapy in gastric cancer. *Nat Commun* 2022; 13(1):5095.

6. Lu L, Ahmed FS, Akin O, et al. Uncontrolled Confounders May Lead to False or Overvalued Radiomics Signature: A Proof of Concept Using Survival Analysis in a Multicenter Cohort of Kidney Cancer. *Front Oncol* 2021; 11:638185.

7. Mukherjee S, Patra A, Khasawneh H, et al. Radiomics-based Machine-learning Models Can Detect Pancreatic Cancer on Prediagnostic Computed Tomography Scans at a Substantial Lead Time Before Clinical Diagnosis. *Gastroenterology* 2022; 163(5):1435-1446.e3.
