# Supplementary figures and images for: Development and validation of a preoperative radiomics-based nomogram to identify patients who can benefit from splenic hilar lymphadenectomy: a pooled analysis of three prospective trials
Source: Int J Surg. 2024 Apr 23;110(7):4053–61. doi: 10.1097/JS9.0000000000001337 (PMC11254245; doi:10.1097/JS9.0000000000001337)

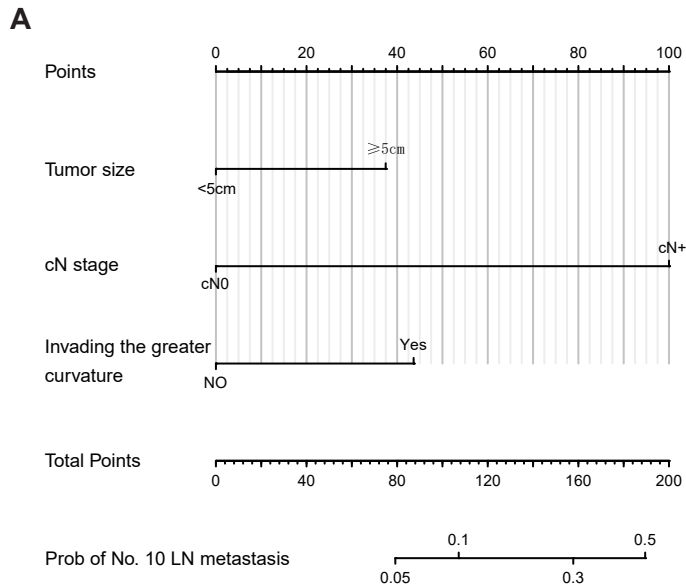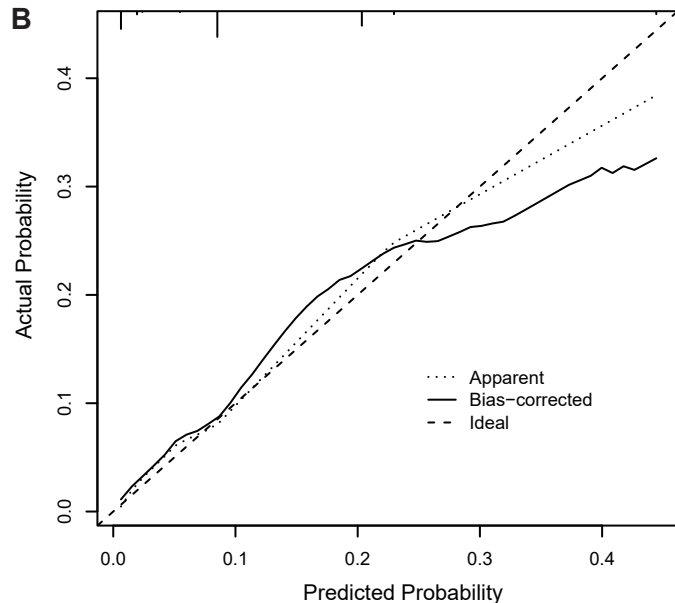

**eFigure 8** (A) Development and (B) performance of clinical models in the training cohort

Supplement: SUPPLEMENTARY MATERIAL [file js9-110-4053-s002.pdf]

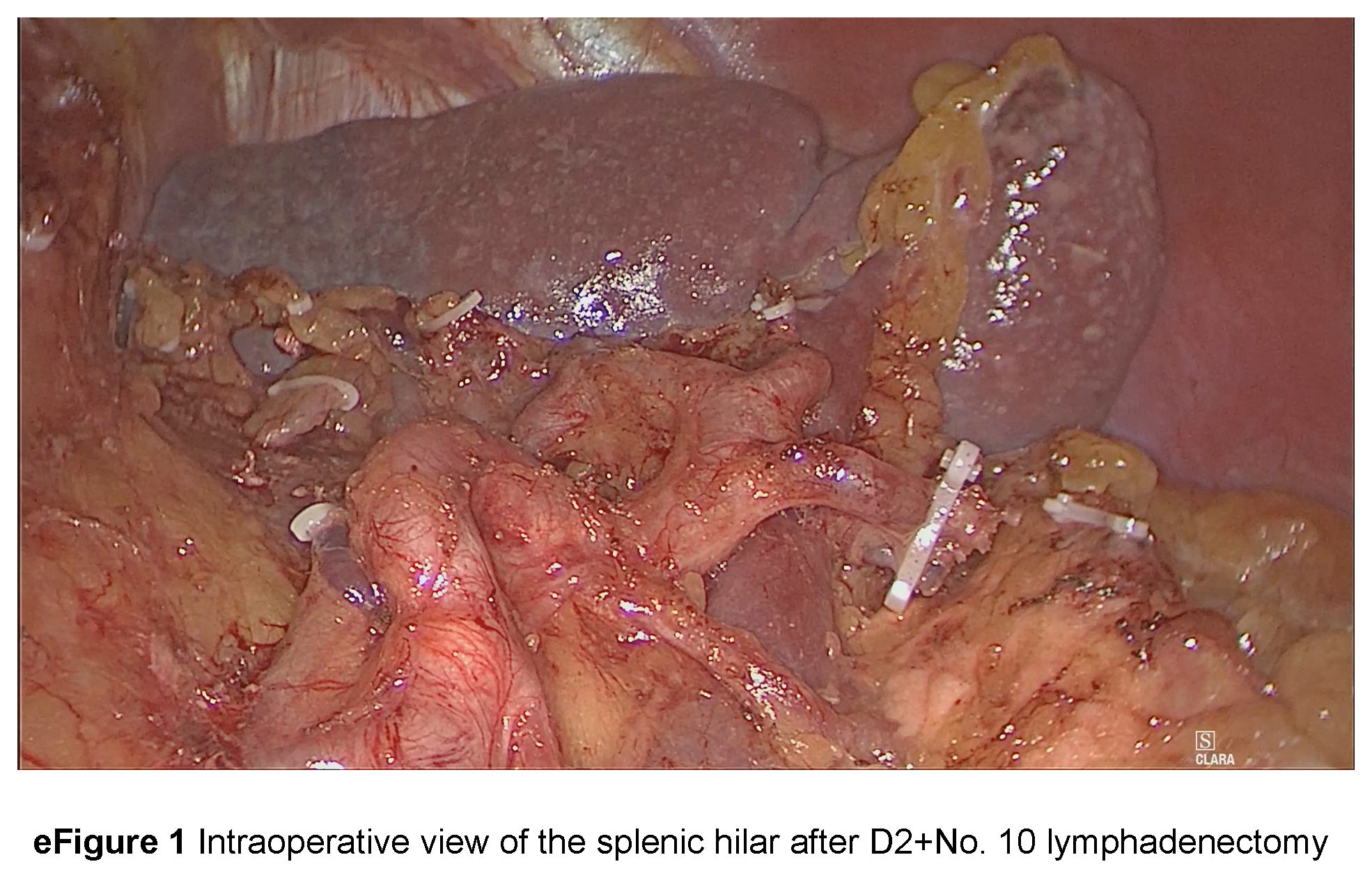

Supplement: SUPPLEMENTARY MATERIAL [file js9-110-4053-s005.jpg]

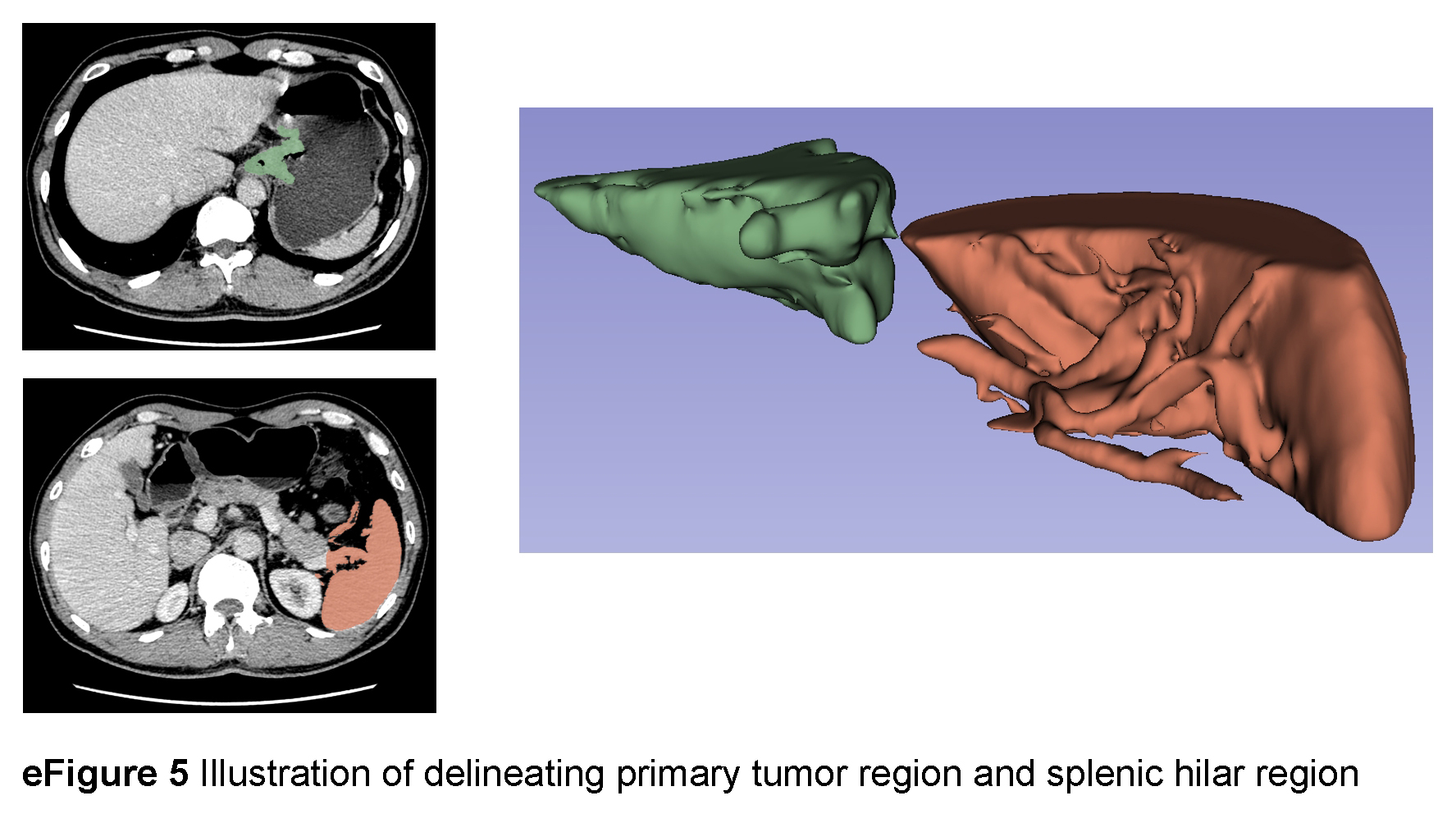

Supplement: SUPPLEMENTARY MATERIAL [file js9-110-4053-s009.jpg]

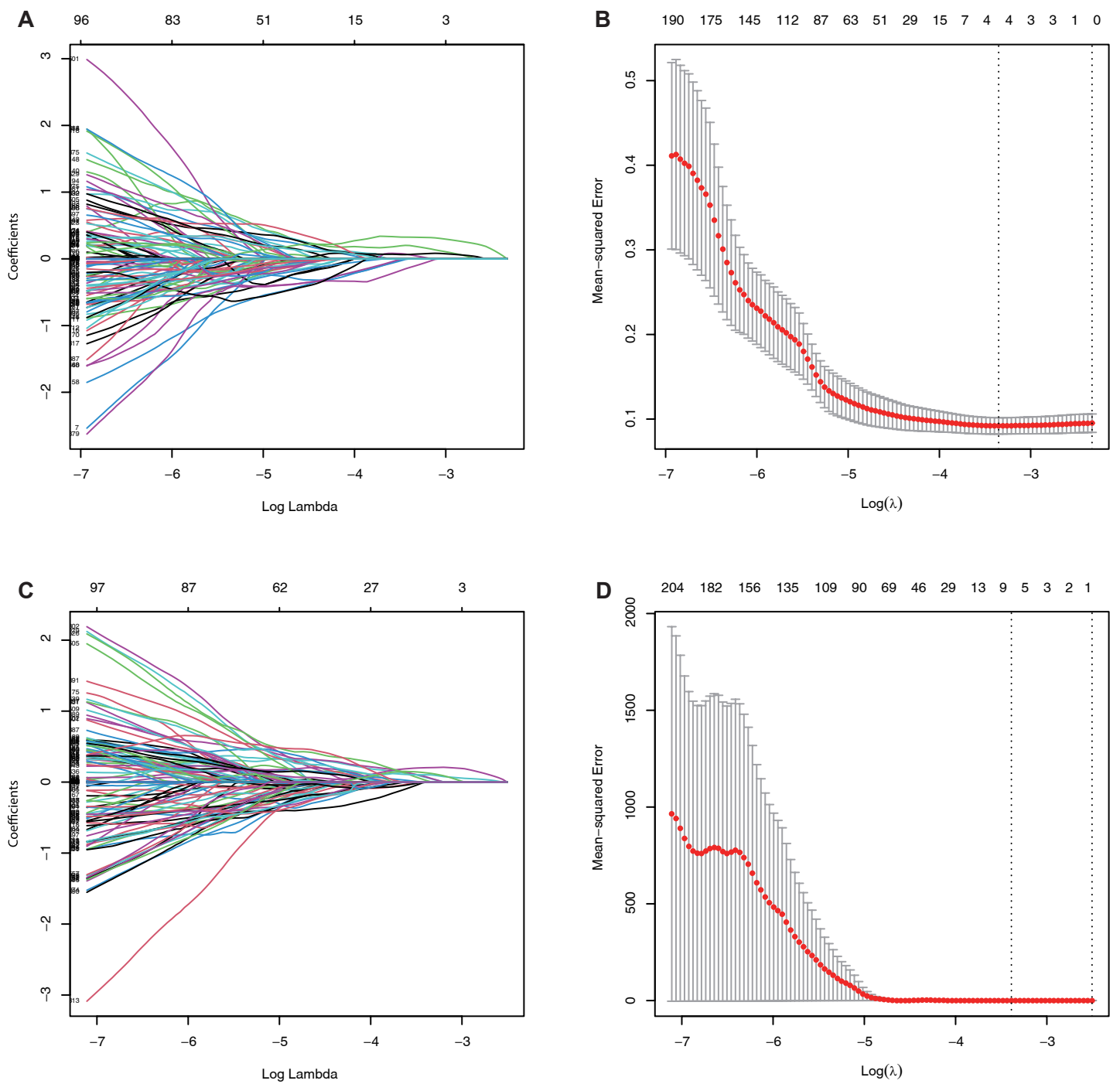

**eFigure 6** LASSO logistic regression on features from the primary tumor (A, B) and splenic hilar (C, D)

Supplement: SUPPLEMENTARY MATERIAL [file js9-110-4053-s010.pdf]
