# Supplementary material for: Development and validation of a preoperative radiomics-based nomogram to identify patients who can benefit from splenic hilar lymphadenectomy: a pooled analysis of three prospective trials
Source: Int J Surg. 2024 Apr 23;110(7):4053–61. doi: 10.1097/JS9.0000000000001337 (PMC11254245; doi:10.1097/JS9.0000000000001337)
Supplement: SUPPLEMENTARY MATERIAL [file js9-110-4053-s004.docx]

| **eTable1 Univariate and multivariate analyses of factors associated with overall survival for patients with No. 10 LN dissection.** | | | | | | |
| --- | --- | --- | --- | --- | --- | --- |
|  | Univariate analysis | | | Multivariate analysis | | |
|  | HR | 95%CI | *p* value | HR | 95%CI | *p* value |
| Age, y |  |  |  |  |  |  |
| ≤60 | 1.000 |  |  | 1.000 |  |  |
| >60 | 1.532 | 1.068-2.199 | 0.021 | 1.674 | 1.154-2.428 | 0.007 |
| Sex |  |  |  |  |  |  |
| Male | 1.000 |  |  |  |  |  |
| Female | 1.063 | 0.730-1.548 | 0.751 |  |  |  |
| Tumor size, cm |  |  |  |  |  |  |
| ≤5 | 1.000 |  |  | 1.000 |  |  |
| >5 | 1.713 | 1.211-2.421 | 0.002 | 0.858 | 0.591-1.245 | 0.420 |
| Histologic type |  |  |  |  |  |  |
| Differentiated | 1.000 |  |  | 1.000 |  |  |
| Undifferentiated | 3.145 | 1.475-6.707 | 0.003 | 1.145 | 0.776-1.688 | 0.495 |
| Lymphovascular invasion |  |  |  |  |  |  |
| Absent | 1.000 |  |  | 1.000 |  |  |
| Present | 2.824 | 1.999-3.989 | <0.001 | 1.250 | 0.836-1.870 | 0.277 |
| Perineural invasion |  |  |  |  |  |  |
| Absent | 1.000 |  |  | 1.000 |  |  |
| Present | 2.395 | 1.692-3.391 | <0.001 | 1.378 | 0.955-1.989 | 0.087 |
| pT stage |  |  |  |  |  |  |
| T1 | 1.000 |  |  | 1.000 |  |  |
| T2 | 1.054 | 0.176-6.309 | 0.954 | 0.709 | 0.116-4.332 | 0.710 |
| T3 | 6.440 | 2.025-20.485 | 0.002 | 2.817 | 0.821-9.661 | 0.100 |
| T4 | 15.119 | 4.743-48.195 | <0.001 | 3.730 | 1.040-13.370 | 0.043 |
| pN stage |  |  |  |  |  |  |
| N0 | 1.000 |  |  | 1.000 |  |  |
| N1 | 2.084 | 0.964-4.507 | 0.062 | 1.315 | 0.587-2.950 | 0.506 |
| N2 | 4.746 | 2.421-9.305 | <0.001 | 2.805 | 1.364-5.771 | 0.005 |
| N3a | 6.775 | 3.570-12.859 | <0.001 | 3.643 | 1.778-7.463 | <0.001 |
| N3b | 16.416 | 8.484-31.762 | <0.001 | 6.611 | 2.977-14.681 | <0.001 |
| Adjuvant chemotherapy |  |  |  |  |  |  |
| Absent | 1.000 |  |  | - |  |  |
| Present | 0.957 | 0.667-1.373 | 0.812 | - |  |  |
| sLNR |  |  |  |  |  |  |
| 0 | 1.000 |  |  | 1.000 |  |  |
| ≤0.4 | 1.150 | 0.561-2.357 | 0.704 | 0.826 | 0.396-1.721 | 0.609 |
| >0.4 | 4.763 | 2.841-7.985 | <0.001 | 1.808 | 1.052-3.105 | 0.032 |
| LN indicates lymph node; sLNR, splenic hilar lymph node ratio | | | | | | |

| **eTable2 Characteristics of patients with No.10 LN metastasis.** | | | |
| --- | --- | --- | --- |
|  | D2 + No.10 Group | | *p* value |
|  | sLNR ≤ 0.4, n = 25 | sLNR ＞ 0.4, n = 21 |  |
| Age, y |  |  | 0.011 |
| ≤60 | 14 (56.0%) | 4 (19.0%) |  |
| >60 | 11 (44.0%) | 17 (81.0%) |  |
| Sex |  |  | 0.695 |
| Male | 18 (72.0%) | 14 (66.7%) |  |
| Female | 7 (28.0%) | 7 (33.3%) |  |
| cT stage |  |  | 0.014 |
| ≤cT2 | 4 (16.0%) | 0 (0.0%) |  |
| cT3 | 12 (48.0%) | 5 (23.8%) |  |
| cT4 | 9 (36.0%) | 16 (76.2%) |  |
| cN stage |  |  | 1.000 |
| cN0 | 1 (4.0%) | 0 (0.0%) |  |
| cN+ | 24 (96.0%) | 21 (100.0%) |  |
| Tumor size, cm |  |  | 0.346 |
| ≤5 | 13 (52.0%) | 8 (38.1%) |  |
| >5 | 12 (48.0%) | 13 (61.9%) |  |
| Histology |  |  | 0.883 |
| Differentiated | 9 (36.0%) | 8 (38.1%) |  |
| Undifferentiated | 16 (64.0%) | 13 (61.9%) |  |
| Cross-sectional part |  |  | 0.735 |
| Non-greater curvature | 19 (76.0%) | 17 (81.0%) |  |
| Greater curvature | 6 (24.0%) | 4 (19.0%) |  |
| Lymphovascular invasion |  |  | 0.845 |
| Absent | 10 (40.0%) | 9 (42.9%) |  |
| Present | 15 (60.0%) | 12 (57.1%) |  |
| Perineural invasion |  |  | 0.246 |
| Absent | 15 (60.0%) | 9 (42.9%) |  |
| Present | 10(40.0%) | 12 (57.1%) |  |
| pT stage |  |  | 0.098 |
| T1 | 1 (4.0%) | 0 (0.0%) |  |
| T2 | 3 (12.0%) | 0 (0.0%) |  |
| T3 | 13 (52.0%) | 8 (38.1%) |  |
| T4 | 8 (32.0%) | 13 (61.9%) |  |
| pN stage |  |  | 0.008 |
| N1 | 5 (20.0%) | 0 (0.0%) |  |
| N2 | 9 (36.0%) | 3 (14.3%) |  |
| N3a | 8 (32.0%) | 8 (38.1%) |  |
| N3b | 3 (12.0%) | 10 (47.6%) |  |
| pTNM stage |  |  | 0.025 |
| II | 6 (24.0%) | 0 (0.0%) |  |
| III | 19 (76.0%) | 21 (100.0%) |  |
| Adjuvant chemotherapy |  |  | 1.000 |
| Absent | 5 (20.0%) | 5 (23.8%) |  |
| Present | 20 (80.0%) | 16 (76.2%) |  |
| LNs indicates lymph nodes; sLNR, splenic hilar lymph node ratio | | | |

| **eTable3 Characteristics of patients in the training and validation cohorts.** | | | |
| --- | --- | --- | --- |
|  | Training cohort, No. (%) | Validation cohort, No. (%) | *p* value |
|  | n = 302 | n = 130 |  |
| Age, y |  |  | 0.059 |
| ≤60 | 134 (44.4%) | 45 (34.6%) |  |
| >60 | 168 (55.6%) | 85 (65.4%) |  |
| Sex |  |  | 0.351 |
| Male | 212 (70.2%) | 97 (74.6%) |  |
| Female | 90 (29.8%) | 33 (25.4%) |  |
| cT stage |  |  | 0.528 |
| ≤cT2 | 64 (21.2%) | 34 (26.2%) |  |
| cT3 | 147 (48.7%) | 59 (45.4%) |  |
| cT4 | 91 (30.1%) | 37 (28.5%) |  |
| cN stage |  |  | 0.664 |
| cN0 | 97 (32.1%) | 39 (30.0%) |  |
| cN+ | 205 (67.9%) | 91 (70.0%) |  |
| Tumor size, cm |  |  | 0.435 |
| ≤5 | 207 (68.5%) | 94 (72.3%) |  |
| >5 | 95 (31.5%) | 36 (27.7%) |  |
| Histology |  |  | 0.181 |
| Differentiated | 114 (37.7%) | 58 (44.6%) |  |
| Undifferentiated | 188 (62.3%) | 72 (55.4%) |  |
| Cross-sectional part |  |  | 0.597 |
| Non-greater curvature | 269 (89.1%) | 118 (90.8%) |  |
| Greater curvature | 33 (10.9%) | 12 (9.2%) |  |
| Lymphovascular invasion |  |  | 0.190 |
| Absent | 182 (60.3%) | 87 (66.9%) |  |
| Present | 120 (39.7%) | 43 (33.1%) |  |
| Perineural invasion |  |  | 0.300 |
| Absent | 172 (57.0%) | 81 (62.3%) |  |
| Present | 130 (43.0%) | 49 (37.7%) |  |
| pT stage |  |  | 0.866 |
| T1 | 41 (13.6%) | 17 (13.1%) |  |
| T2 | 24 (7.9%) | 13 (10.0%) |  |
| T3 | 157 (52.0%) | 69 (53.1%) |  |
| T4 | 80 (26.5%) | 31 (23.8%) |  |
| pN stage |  |  | 0.492 |
| N0 | 96 (31.8%) | 38 (29.2%) |  |
| N1 | 56 (18.5%) | 21 (16.2%) |  |
| N2 | 54 (17.9%) | 28 (21.5%) |  |
| N3a | 59 (19.5%) | 32 (24.6%) |  |
| N3b | 37 (12.3%) | 11 (8.5%) |  |
| pTNM stage |  |  | 0.521 |
| I | 49 (16.2%) | 23 (17.7%) |  |
| II | 98 (32.5%) | 35 (26.9%) |  |
| III | 155 (51.3%) | 72 (55.4%) |  |
| Adjuvant chemotherapy |  |  | 0.425 |
| Absent | 95 (31.5%) | 46 (35.4%) |  |
| Present | 207 (68.5%) | 84 (64.6%) |  |

| **eTable4 Description of the selected radiomic features** | | | | |
| --- | --- | --- | --- | --- |
| Region | Features | Group | Image type | Coefficient |
| primary tumor | Large Dependence High Gray Level Emphasis | gldm | *log-sigma-2-0-mm-3D* | 0.019888981 |
|  | Large Area High Gray Level Emphasis | glszm | *X_LHH_* | 0.016135357 |
|  | lmc2 | glcm | *X_HLL_* | -0.008789638 |
|  | Kurtosis | firstorder | *X_HHH_* | 0.041629276 |
| Splenic hilar | Inverse Variance | glcm | *log-sigma-1-5-mm-3D* | 0.0260577 |
|  | firstorder | Kurtosis | *log-sigma-2-5-mm-3D* | 0.0248952 |
|  | Skewness | firstorder | *X_LHH_* | 0.03872172 |
|  | Cluster Prominence | glcm | *X_HHL_* | 0.06053678 |
|  | Kurtosis | firstorder | *X_HHH_* | 0.03336477 |

| **eTable5 Incidence of lymph node metastasis, 5-year survival rates, and therapeutic value index for No.10 LN.** | | | |
| --- | --- | --- | --- |
| Different groups | Patients with SPSHL | | |
|  | Metastatic incidence (%) | 5-year OS of patients with No.10 LN metastasis (%) | Therapeutic value index |
| LMB group | 0.6 | 100.0 | 0.6 |
| MMB group | 29.3 | 61.3 | 18.0 |
| HMB group | 85.0 | 5.9 | 5.0 |
| SPSHL indicates spleen-preserving splenic hilar lymphadenectomy; OS, overall survival; LMB, low splenic hilar lymph node metastasis burden; MMB, middle splenic hilar lymph node metastasis burden; HMB, high splenic hilar lymph node metastasis burden. | | | |
