# Supplementary material for: Development and validation of a preoperative radiomics-based nomogram to identify patients who can benefit from splenic hilar lymphadenectomy: a pooled analysis of three prospective trials
Source: Int J Surg. 2024 Apr 23;110(7):4053–61. doi: 10.1097/JS9.0000000000001337 (PMC11254245; doi:10.1097/JS9.0000000000001337)
Supplement: SUPPLEMENTARY MATERIAL [file js9-110-4053-s007.pdf]

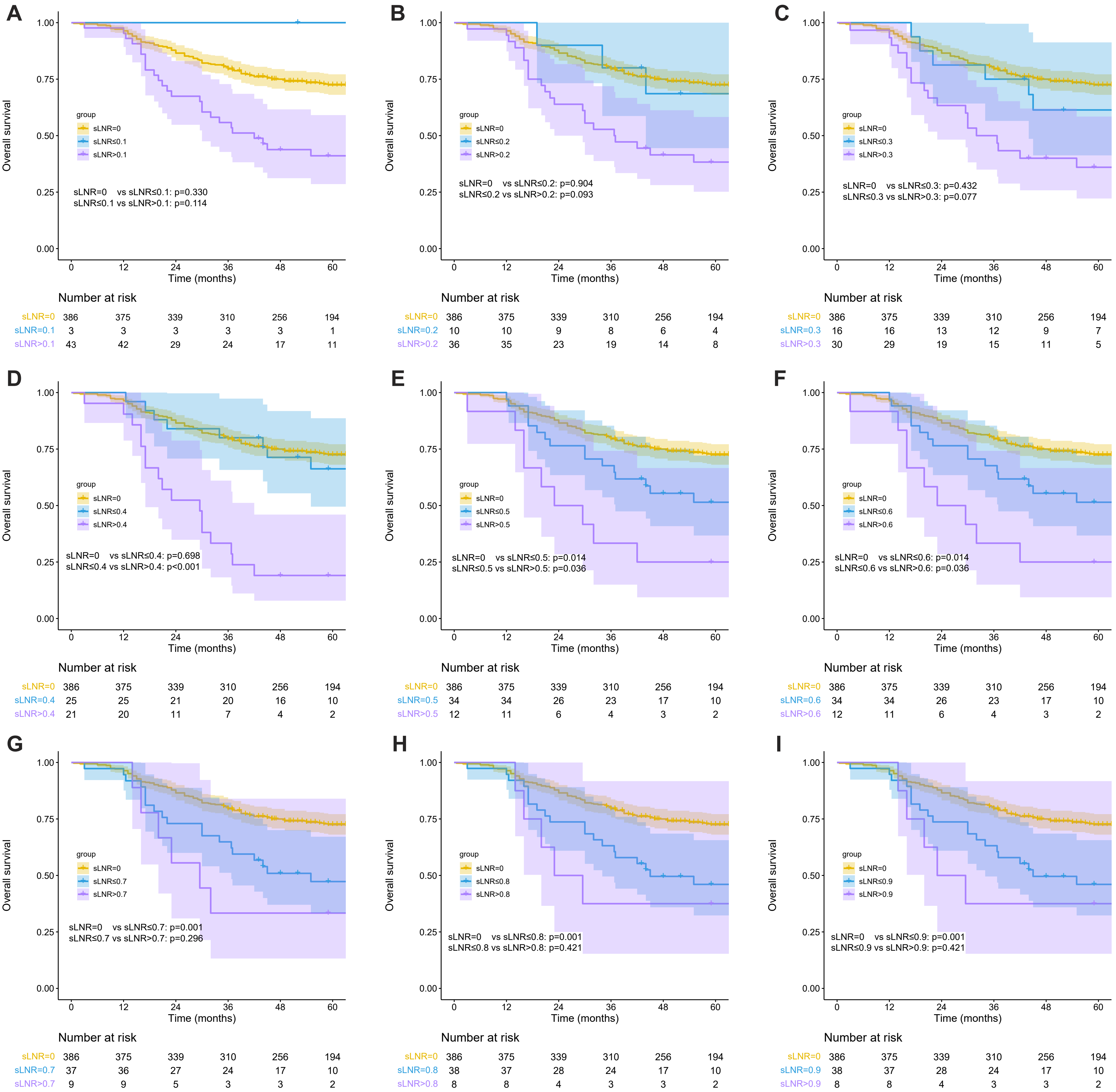

eFigure 3 Stepwise analysis of optimal cut-off value (from 0.1 to 0.9) for splenic hilar lymph node ratio (sLNR) based on overall survival
