# Supplementary material for: Development and validation of a preoperative radiomics-based nomogram to identify patients who can benefit from splenic hilar lymphadenectomy: a pooled analysis of three prospective trials
Source: Int J Surg. 2024 Apr 23;110(7):4053–61. doi: 10.1097/JS9.0000000000001337 (PMC11254245; doi:10.1097/JS9.0000000000001337)
Supplement: SUPPLEMENTARY MATERIAL [file js9-110-4053-s008.pdf]

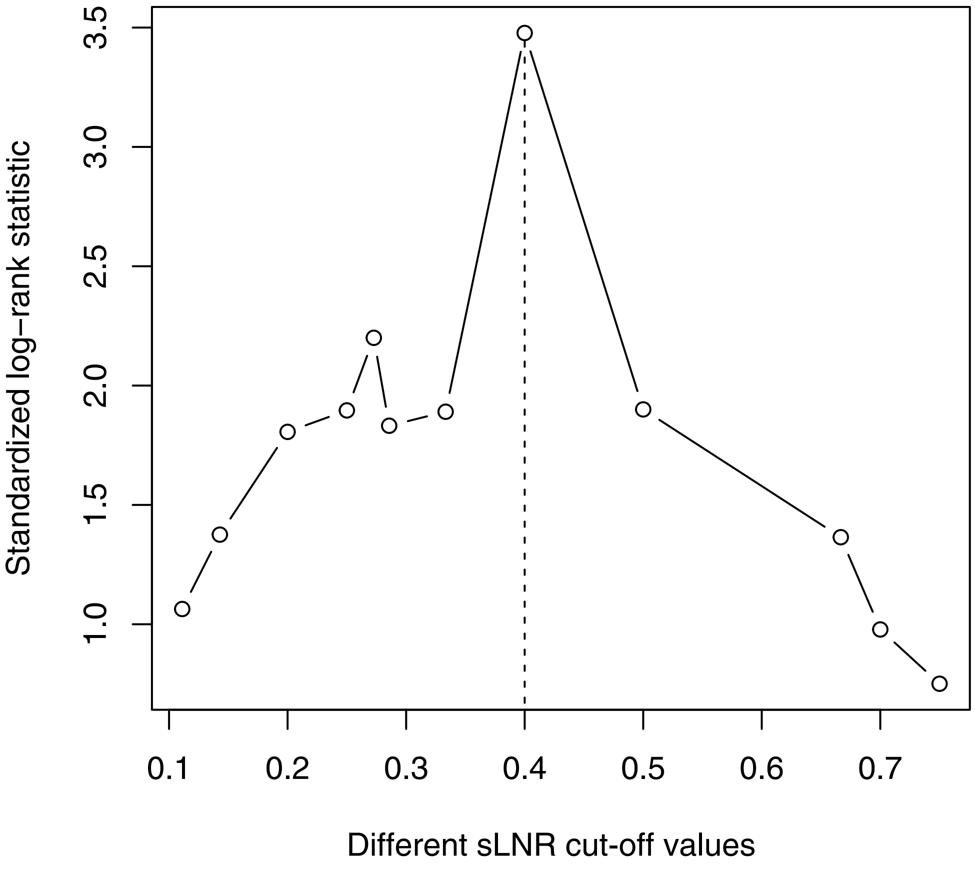

eFigure 4 Statistical differences in overall survival between the two groups at different sLNR cutoff values (log-rank methods)
